# Heat Stress-Induced Dysbiosis of Porcine Colon Microbiota Plays a Role in Intestinal Damage: A Fecal Microbiota Profile

**DOI:** 10.3389/fvets.2022.686902

**Published:** 2022-03-01

**Authors:** Canying Hu, Yadnyavalkya Patil, Dongliang Gong, Tianyue Yu, Junyu Li, Lianyun Wu, Xiaoxi Liu, Zhichao Yu, Xinbing Ma, Yanhong Yong, Jinjun Chen, Ravi Gooneratne, Xianghong Ju

**Affiliations:** ^1^Department of Animal Science, Agricultural College, Guangdong Ocean University, Zhanjiang, China; ^2^Shenzhen Institute of Guangdong Ocean University, Shenzhen, China; ^3^Department of Wine, Food and Molecular Biosciences, Faculty of Agriculture and Life Sciences, Lincoln University, Lincoln, New Zealand; ^4^Department of Veterinary Medicine, College of Agriculture, Guangdong Ocean University, Zhanjiang, China

**Keywords:** heat stress, colonic microbiome, inflammation in the colon, pigs, mice

## Abstract

The pathological mechanisms of gastrointestinal disorders, including inflammatory bowel disease (IBD), in pigs are poorly understood. We report the induction of intestinal inflammation in heat-stressed (HS) pigs, fecal microbiota transplantation from pigs to mice, and explain the role of microorganisms in IBD. 24 adult pigs were subjected to HS (34 ± 1 °C; 75–85% relative humidity for 24h) while 24 control pigs (CP) were kept at 25 ± 3°C and the same humidity. Pigs were sacrificed on days 1, 7, 14, 21. Colonic content microbiome analyses were conducted. Pseudo-germ-free mice were fed by gavage with fecal microbiota from HS-pigs and CP to induce pig-like responses in mice. From 7 d, HS-pigs exhibited fever and diarrhea, and significantly lower colonic mucosal thickness, crypt depth/width, and goblet cell number. Compared with each control group, the concentration of cortisol in the peripheral blood of HS pigs gradually increased, significantly so on days 7, 14, and 21 (P < 0.01). While the concentration of LPS in HS pigs' peripheral blood was significantly higher on days 7, 14 (P < 0.01), and 21 (P < 0.05) compared with that of the control group. The colonic microbiome composition of HS-pigs was different to that of CP. By day 14, opportunistic pathogens (e.g., *Campylobacterales*) had increased in HS-pigs. The composition of the colonic microbiome in mice administered feces from HS-pigs was different from those receiving CP feces. *Bacteroides* were significantly diminished, *Akkermansia* were significantly increased, and intestinal damage and goblet cell numbers were higher in mice that received HS-pig feces. Moreover, we verified the relevance of differences in the microbiota of the colon among treatments. Heat stress promotes changes in gut microbiome composition, which can affect the colonic microbial structure of mice through fecal microbiota transplantation; the molecular mechanisms require further investigation. This study enhanced our understanding of stress-induced inflammation in the colon and the increase in diarrhea in mammals subjected to prolonged HS. Our results provide useful information for preventing or ameliorating deficits in pig production caused by prolonged exposure to high temperatures.

## Background

Heat stress (HS) refers to heat received in excess of that which the body can tolerate, without physiological impairment. The recent increase in global warming has revealed the harmful effects of heat stress on human health, animal welfare, and animal husbandry production (1). Pigs are more sensitive to HS, compared to other livestock animals, because they produce high metabolic heat, there is quick fat deposition, and lack of functional sweat glands (2). HS conditions in pigs decrease the food intake, body weight (BW) gain, and meat quality. These together can cause severe losses, as has been evident for the US swine industry, which lost over $300 million annually to HS in 2010 (3). HS response can markedly reduce post-absorption of carbohydrate, lipid, and protein metabolism in pigs independently of reduced feed intake (4–6), which were associated with intestinal hyperpermeability and swine reproduction (7). Research by Santos et al. (8) and Campos et al. (9) show considerable reduction in growth performance and activated inflammatory responses in growing pigs in high-temperature conditions. Moreover, HS decreased the skeletal muscle fatty acid oxidation and metabolic flexibility in pigs (10).

Inflammatory bowel disease (IBD) affects >3.1 million people in the United States and 2.5 million in Europe annually and its incidence is increasing worldwide, especially in East and South Asia (11, 12). IBD is characterized by chronic intestinal inflammation and lesions triggered by genetic predisposition and environmental factors (13). Various stressors such as emotional distress, hypothermia, and hyperthermia may perturb the gut-brain axis, induce inflammation, and aggravate or precipitate gastrointestinal disorders such as Crohn's disease and ulcerative colitis. It is reported that the alpha diversity of the microbiome and the *Firmicutes* / *Bacteroidetes* of intestinal microbiome in IBD patients were decreased, and the number of *Proteobacteria* was increased (14). Studies have shown that HS increased intestinal mucosal inflammation and damage in pigs, chickens, and rats (15). Nevertheless, the pathophysiological mechanisms of these processes are unknown. Global mean temperature and relative humidity have increased by ~10.1 and 1.8% respectively since the 1880s (16). As summertime temperatures and relative humidity levels increase with global climate change, the incidence and severity of thermal stress-related illness are expected to rise as well (17).

Heat shock proteins (HSPs) are a type of stress protein that exist in prokaryotic and eukaryotic organisms (18). When the body is in a high-temperature environment, the cells in the body will synthesize such proteins to protect the body. HSP70 and HSP27 has been studied most extensively (19, 20). Heat stress enhances the expression of HSP70 and the concentration of cortisol in mammals (21–23). In heat-stressed animals, dermal blood vessels dilate in order to maximize peripheral heat loss. Consequently, the blood supply to the intestine is reduced, which decreases the intestinal oxygen supply and triggers localized inflammation and intestinal damage (24). Increased intestinal permeability may cause intestinal Lipopolysaccharide (LPS), in which intestinal microbes play a vital role (25, 26). We propose that reduced intestinal blood and oxygen supply are not the only factors responsible for stress-induced inflammation in the colon. Other stressors such as psychosocial events, hypothermia, and hyperthermia may also promote the inflammation in the colon of IBD development (27, 28). HSPs have been described as associated with the gut intestinal mucosa and its function. Liu et al., reported an association between the cytoprotective HSP70 and the relative abundance of *Lactobacillus* spp, and modifications to bacterial colonization during early life functions to control the intestinal architecture and function. (29). Furthermore, long-term site- and diet-specific effects are observed in the major immune components that serve to control intestinal homeostasis (30).

The mammalian gastrointestinal tract hosts ~10^14^ microorganisms comprising 500–1,000 unique species, which form synergistic mutualisms with their host (31–34). Co-evolution of gut microorganisms with their hosts has resulted in the role specialization of different microbes in digestion, nutrient utilization, toxin removal, pathogen protection, and regulation of endocrine and immune systems (35–37). A healthy intestinal microbial community is diverse, stable, resistant to minor changes, and resilient (38). Human and mouse studies have shown that gut dysbiosis (i.e., disequilibrium of the microbial community) is associated with various acute and chronic inflammatory conditions, bowel diseases, metabolic syndromes, and diabetes (39). Gut dysbiosis and reduced complexity of the gut microbial ecosystem are common symptoms in patients with Crohn's disease or ulcerative colitis. Nevertheless, it is not known whether these alterations are causes or consequences of these diseases (40).

Pigs and humans have anatomical, physiological, and immunological similarities. Thus, studies of the immunological mechanisms in pigs could be reproducible in humans. Moreover, pigs have similar susceptibilities and clinical manifestations in response to pathogens causing certain human intestinal disorders (41, 42). Xiao et al. reported that the homology between human and pig microbiomes is low at the gene level but significantly higher at the level of Kyoto Encyclopedia of Genes and Genomes (KEGG) orthology functions. Xiao et al. also identified greater similarity between human and pig microbiomes than that between human and mouse microbiomes. They reported that approximately 96% of the functional pathways described in the human gut microbiome resemble those in pigs (43).

Fecal microbial transplantation (FMT) can successfully treat relapsed *Clostridium difficile* infections that are resistant to antibiotics; FMT seems to be beneficial for some patients for other gastrointestinal diseases such as IBD. The effects of FMT may be through specific microorganisms or their active products. The mechanisms behind adjusting the intestinal microbiota to treat diseases remain to be explored (44). The aim of this study was to elucidate the role of intestinal microbial composition in inflammation in the colon development in heat-stressed pigs. In order to explore whether microbial changes are the cause of inflammation, the FMT test of mice was designed. The mice were used to build a pseudo-sterile model after feed combination antibiotics and then fed feces from different groups of heat-stressed pigs to explore changes in the gut microbiota and its contribution to inflammation.

## Methods

### Animals and Management

The experimental protocols involving the management and care of pigs and mice were approved by the Animal Care and Use Committee of Guangdong Ocean University, Zhanjiang, China (Permit No. 206-1108).

### Pigs

Forty-eight pigs (Luchuan sows × Duroc boars; 24 males and 24 females), each weighing 15 ± 2 kg, were housed in two animal rooms at the Animal Hospital of Guangdong Ocean University, Zhanjiang, China. The pigs were randomly divided into two animal-room groups (control and heat stress treatment). Each group had six males and six females of similar body weight. There were four replicates (day 1, 7, 14, and 21) of three per group (n=3). The animals were maintained for 2 weeks at 20 ± 2°C and relative humidity (RH) of 75–85% to acclimatize them to the environment. The photoperiod was maintained under 12:12 h (light: dark) over the adaptation and trial periods. Throughout the study, the pigs were fed a complete feed formula in the morning, afternoon, and evening with ~6 h intervals between feedings. Drinking water was freely available. To minimize acute heat stress, the animal facility was gradually warmed over a 7d period. The control animals were exposed to 25 ± 3°C, and the HS animals were subjected to 34 ± 1°C, at 75–85% RH and lasted 21 d.

### Mice

Pseudo germ-free animals (SPF grade) were prepared by feeding mice (*n* = 130) with a mixture of vancomycin (200 mg kg^−1^), metronidazole (200 mg kg^−1^), and neomycin (200 mg kg^−1^) for 5 d consecutively (45). Throughout the study period, all mice were maintained under a 12:12 h (light: dark) photoperiod and an air circulation cycle under the Exhaust Ventilated Closed-System Cage Rack. All the mice were kept and operated in the strictly sterile environment during the test, all the mice were maintained under an air circulation cycle under the exhaust ventilated closed-system cage rack (Suzhou Guchuang Educational Experimental Animal Cage Co., Ltd., IVC cages sealed, and placed in microisolators), they had *ad libitum* access to autoclave-sterilized feeds and water. All the materials and equipment for test were autoclaved.

Ten mice were controls (BC group) and received feed and *ad libitum* water. The other mice (*n* = 120) were divided into four groups of 30 each. They were administered FMT after pig feces collection on days 1, 7, 14, and 21. Each group was further subdivided into three treatment groups of 10 mice each. They were administered intragastric infusions of either phosphate-buffered saline (PBS group), a 0.5 mL mixture of control pig feces homogenized in PBS (CF group), or a 0.5 mL mixture of HS pig feces homogenized in PBS (HF group). All mice were sacrificed by dislocating the neck after gavage for 7 d.

### Health and Safety

All our experimental procedures were conducted according to the universally accepted codes of laboratory practice and/or as per manufacturer's instructions and followed all health and safety guidelines advocated by them.

### Sample Collection

All pigs were observed for diarrhea and body weight gain. Fecal consistency and color were recorded daily and scored as shown in Table 1. The Diarrhea Index was calculated according to the score for six pigs sacrificed at each sampling time.

**Table 1:** Diarrhea index^#^.

**Diarrhea score**	**Degree of hardness/softness of feces**
1	Hard, dry, and blocky
2	Hard
3	Soft
4	Soft and mushy
5	Watery
6	Watery, yellow, foamy

# *Based on the method of Rossi et al. (46)*.

Diarrhea Index = sum of scores / number of pigs (46).

The pigs were sacrificed by electric shock fainting and bloodletting, and each segment of the colonic tissue was separated by abdominal operation. The sampling position was as consistent as possible. After the separation of adipose and mesentery of colonic tissue, the colonic tissues (all are close to the cecum) of each segment were cut and stored at −80°C. Next, 1-cm segments of the colon were cut and soaked in 10% formaldehyde solution for fixation. The other tissues were cleaned with sterile phosphate-buffered saline (PBS), cut into small pieces, packed in labeled sample bags, and quickly frozen in liquid nitrogen. After collecting, the tissue samples and intestinal contents were transferred to −80°C for cryopreservation. After FMT administration, mice (*n* = 30 per collection day) were sacrificed on day 7. Colonic feces and colon tissue samples were collected and immediately stored at −80°C until subsequent analyses.

### Morphological Observations

Colonic tissue was fixed in buffered formalin (10% v/v) and stained with H&E for histopathological examination. Hydrated colonic tissue sections were treated with amylase at 37°C for 1 h, rinsed under running water for 10 min, and stained with periodic acid solution at room temperature (25 ± 2C) for 7 min according to the instructions for the Glycogen D-PAS Staining Kit (Leagene Biotechnology, Beijing, China). The tissue sections were rinsed with tap water, immersed in Schiff's reagent in the dark for 15 min, and rinsed with tap water for 10 min to remove the stain. The sections were dehydrated with an alcohol concentration gradient (75%, 85%, 95%, and then 100%), cleared of alcohol with xylene, and sealed with neutral gum. Image-Pro Plus v. 6.0 (Media Cybernetics Inc.) was used to evaluate the number of goblet cells in mucosal tissue area of the colonic mucosa (47). Colonic histological damage is assessed by using a scoring system that slices were evaluated by the experienced pathologist in a blinded manner and histological scores were assessed based on the following parameters according to previous research: inflammation (0–3), epithelial defects (0–3), crypt atrophy (0–3), dysplasia / neoplasia (0–3), and the area affected by dysplasia (0–3) (48).

### Microbial Genomic Sequencing

After the animal were sacrificed, the designated part of the colon (all are close to the cecum) is ligated with both ends to collect the contents, and 2 g of the colon content from pigs (*n* = 3 in each group) /mice (*n* = 3 in each group) was collected to Microbiology sequencing. Total genomic DNA was extracted from the samples with a QIAamp DNA Stool Mini Kit (Qiagen, Hilden, Germany) according to the manufacturer's instructions. DNA purity were evaluated on 1% agarose gel. The quantity of DNA was determined with a NanoDrop 1000 spectrophotometer (Thermo Fisher Scientific, Waltham, MA, USA) after calibration with sample solvents and the DNA was diluted to 1 ng μL^−*l*^ with sterile water. The V3–V4 distinct regions of the 16S rRNA genes were amplified with specific barcoded primers (49, 50). The PCR reactions were performed in triplicate in a total volume of 25 μL consisting of 1 μL of each the primers (5 μM), 10 μL of 10 ng DNA template, 4 μL of 1×FastPfu buffer, 1 μL of 2.5 mM dNTPs, 0.4 μL FastPfu polymerase, and 7.6 μL nuclease-free water. The PCR procedure was as follows: initial denaturation at 94°C for 5 min, 30 cycles at 94°C for 50 s, 55°C for 30 s, 72°C for 50 s, and a final extension at 72°C for 6 min. The PCR products were purified with an AxyPrep DNA Gel Extraction Kit (Axygen Scientific Inc., Union City, CA, USA). Amplicons from all samples were sent to a commercial company (Biomarker Technologies Corporation, Beijing, China) for sequencing on an Illumina HiSeq 2500 platform (Illumina, San Diego, CA, USA).

### Microbial Genomic Analyses

Species classification information corresponding to each operational taxonomic unit (OUT) was obtained by comparing the representative OTU sequences with the microbial reference database. After rarefying OUT samples (sample size < minimum of samples), sample community compositions were calculated at the phylum, class, order, family, genus, and species levels and generated in QIIME (Version 2.0.0). GraphPad Prism v. 6.0c (GraphPad Software, San Diego, CA, USA), R (v. 3.0.3), Metastats, and STAMP (Statistical Analysis of Metagenomic Profiles) were used for the statistical analyses. The weighted UniFrac distances among the samples and groups were statistically compared by analysis of similarities using the “vegan” package of R (v. 3.0.3). Mothur was used to analyze the Alpha diversity index of each sample, including the Shannon and Simpson indexes (51). QIIME software for Beta diversity analysis was used to compare the similarity of different samples in terms of species diversity; the binary algorithm was used to calculate the sample distance. We used R to draw Shannon index curves, rank abundance curves, species accumulation curves, PCoA plots, and a heatmap of sample distances. In the univariate analysis of gut microbiota and predicted KEGG biochemical pathways for each group, a one-way ANOVA with Bonferroni's multiple comparison test was performed to compare the alpha diversities among groups. Metastats identified differentially abundant phyla, genera, classes, and species in the groups. Significant differences between groups were identified by the LEfSe (line discriminant analysis effect size) method. PICRUST software was used to compare the species composition information obtained from 16S sequencing data to determine the functional gene composition of the samples, and analyze the functional differences between different samples or groups. At the genus level, the G-TEST and Fisher test were used to detect differences in the abundance of species between samples. Pairwise *t-*tests were used to detect differences between groups, assuming a *P-*value threshold for significance of 0.05. Random forest analysis was used to test the importance of species. RDA (Redundancy analysis), CCA (Canonical Correspondence analysis), and mantel test analysis in R language vegan package were used to analyze and map the relationships between treatment temperature, time, and diarrhea index and changes in microbial microbiota.

### Western Blotting

To measure the responses of the critical heat shock response in the colonic tissues, total protein was extracted with radioimmunoprecipitation assay (RIPA) lysis buffer (Beyotime Biotechnology, Shanghai, China; Cat # P0013B) and the nuclear and cytoplasmic protein fractions were extracted with NE-PERTM nuclear and cytoplasmic extraction reagents (Thermo Fisher Scientific, USA; Cat # 78833), respectively. The total protein concentration was determined using a BCA (bicinchoninic acid) protein assay kit (CWBIO, Beijing, China; Cat # CW0014). Equal amounts of protein lysate were separated by sodium dodecyl sulfate-polyacrylamide gel electrophoresis (SDS-PAGE) and electro-transferred to nitrocellulose membranes (Merck Millipore, Burlington, MA, Germany; Cat # 3010040001). The membranes were blocked for 1 h with 5% skimmed milk powder and incubated with the primary antibody Anti-HSP27 (Abcam, Burlingame, CA, USA; Cat # ab2790), HSP70 (Abcam; Cat # ab5439), and β-actin (Beyotime Biotechnology; Cat # AF5003) overnight at 4°C. The blots were incubated at 4°C for 2 h with a corresponding secondary antibody conjugated to horseradish peroxidase (HRP). HRP-conjugated anti-rabbit IgG and anti-mouse IgG were obtained from Cell Signaling Technology (Beyotime Biotechnology; Cat # A0408 & A0412). Positive bands were visualized by enhanced chemiluminescence (ECL; Tanon, China; Cat # 180-5001). The band intensities were semi-quantitatively analyzed by densitometry with a Gel-Pro Analyzer v. 4.0 (Meyer Instruments, Houston, TX, USA).

### Enzyme-Linked Immunosorbent Assay (ELISA)

Serum was stored at −80°C prior to analysis. Thawed and centrifuged (3,000 ×g at 4 °C for 15 min) serum supernatant was used in following analysis. Serum biochemical markers were measured using ELISA kits for porcine Cortisol (COR; MeiMianBio, Wuhan, China; Cat # MM039901) and Lipopolysaccharide (LPS; MeimianBio; Cat # MM3636801) according to the manufacturer's protocol. The plates were read using a microplate reader (BioTek Instruments Inc., Winooski, VT, USA) at a wavelength of 450 nm. A standard curve for each of the biochemical markers was used to estimate the concentration.

## Results

### Clinical Signs in Pigs

**Figure 1:**
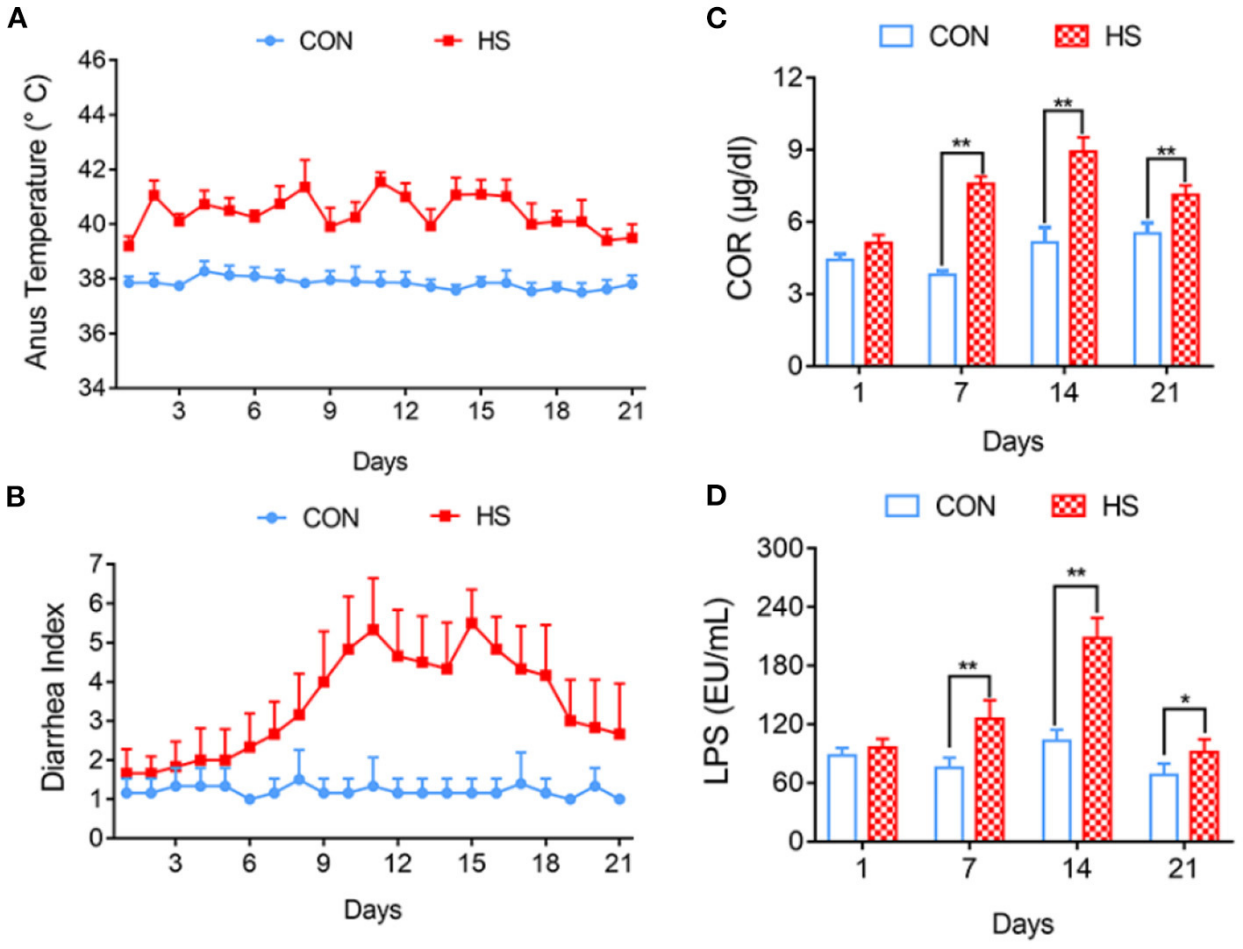
Effects of heat stress on pig anus temperature and growth performance. **(A)** Anus temperature; **(B)** Diarrhea Index; **(C)** Concentration of COR in Peripheral blood; **(D)** Concentration of ET in Peripheral blood. *p < 0.05, **p < 0.01 compared with each group.

The average rectal temperature on day 3 of HS group was 40.46°C, which was 2.84°C higher than that of the control group (Figure 1A). The Diarrhea Index (DI) for the control group was <3 and the pigs showed no symptoms of diarrhea. In the HS group, diarrhea was observed on day 7 and gradually increased thereafter. The DI from days 8 to 19 was >3. Diarrhea was most severe on days 11 and 15 and the DI scores were 5.5 and 5.6, respectively (Figure 1B).

### Effect of HS on COR and LPS Concentrations of Peripheral Blood in Pigs

Compared with each control group, the COR concentration in the peripheral blood of the HS group gradually increased and was significantly different on days 7, 14, and 21 (*P* < 0.01) (Figure 1C). The concentration of LPS in the pigs' peripheral blood, was significantly higher in the HS group on days 7, 14 (*P* < 0.01), and 21 (*P* < 0.05) compared with that of each control group (Figure 1D).

### Relative Expression of Heat Shock Proteins in Pigs' Colonic Tissues After HS

**Figure 2:**
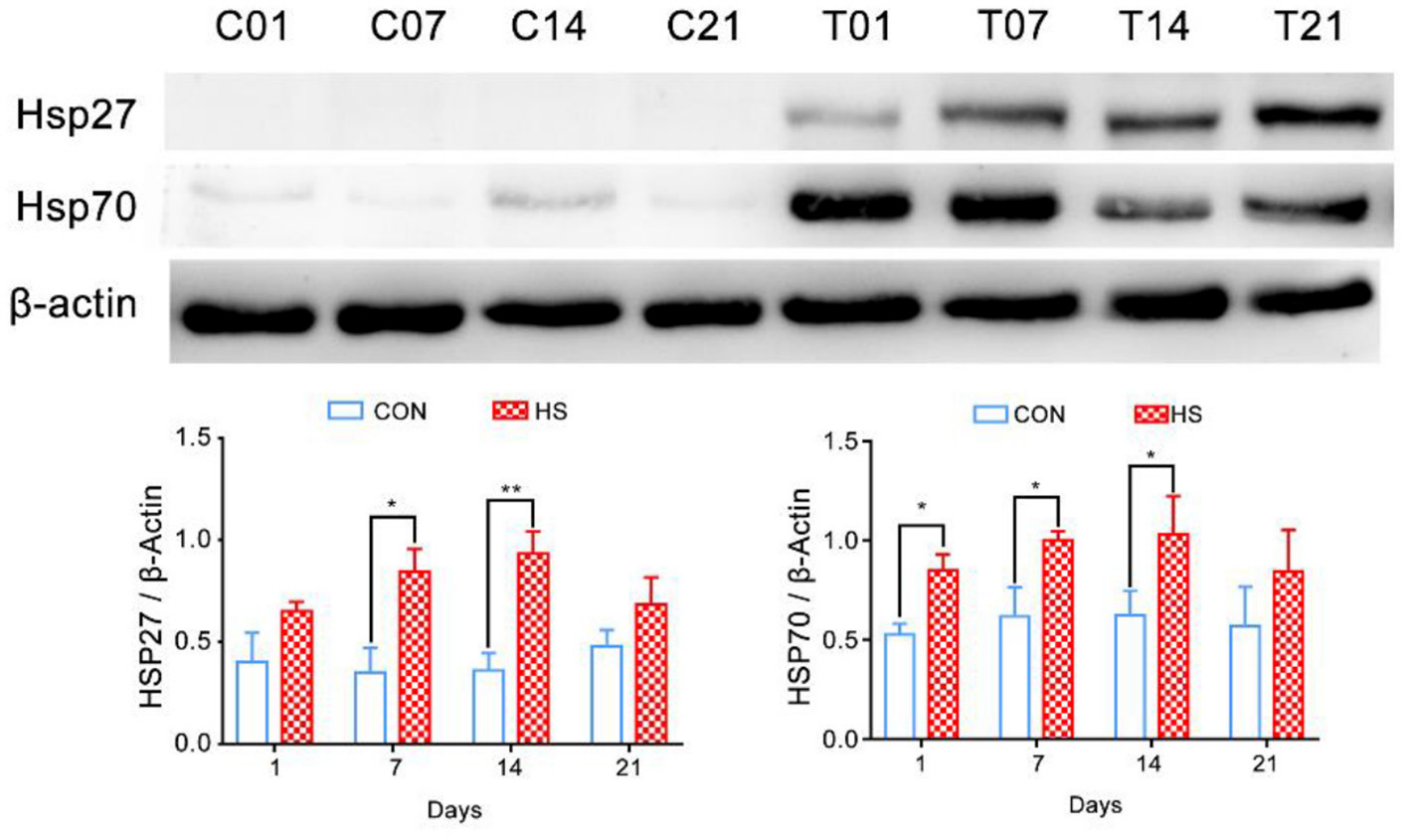
Effect of heat stress on relative expression of heat shock proteins in pigs' colonic tissue. *p < 0.05, **p < 0.01 compared with each group.

The relative expression of HSP27 and HSP70 in pigs' colonic tissue were significantly higher in HS pigs on days 7 and 14 (*P* < 0.05) compared with that of each control group; however, at day 21, the decreased of HSP27 and HSP70 were not significantly (Figure 2).

### Histopathology of Colonic Mucosa in Pigs

**Figure 3:**
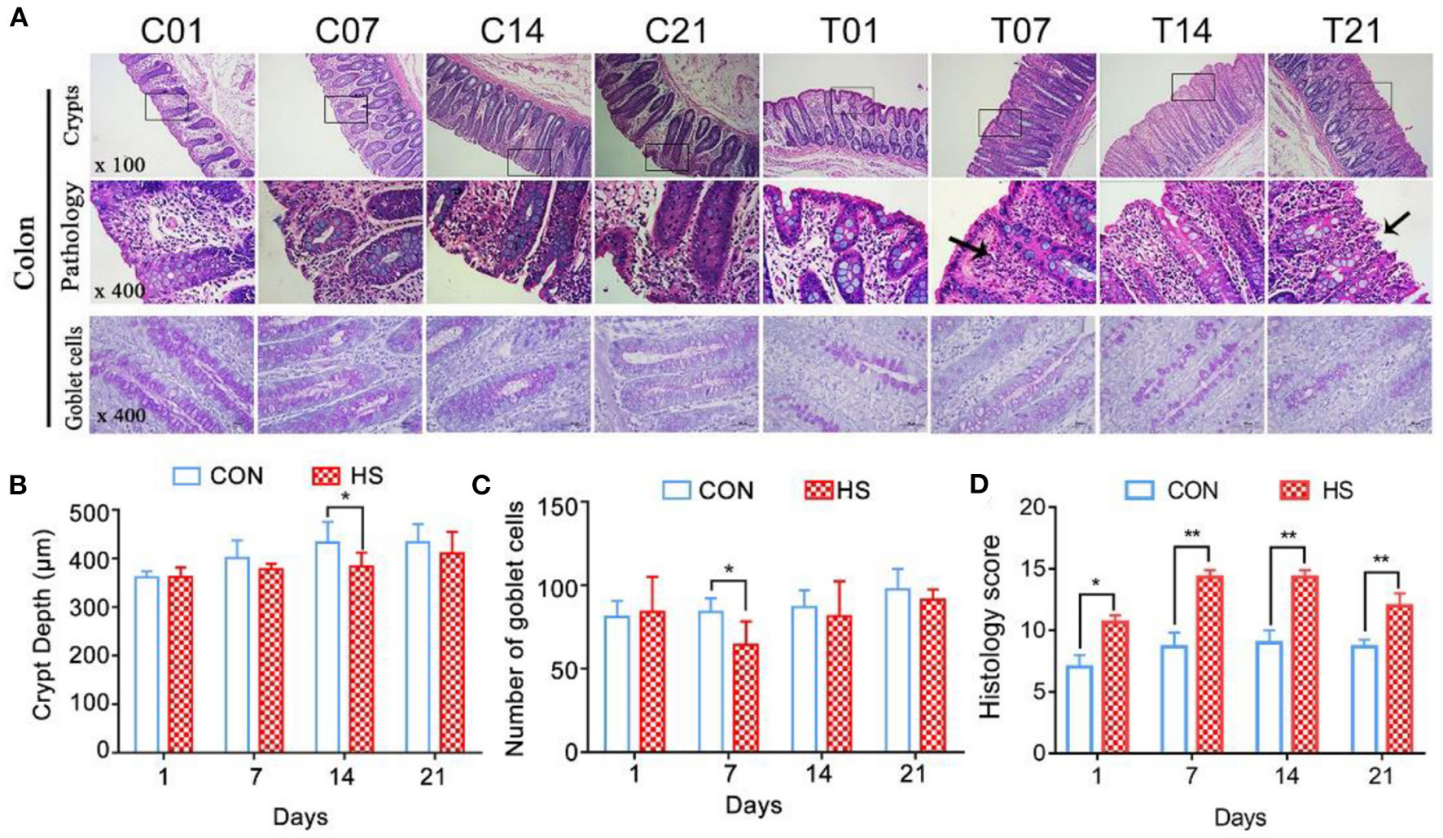
Effects of heat stress on colonic mucosal structure in pigs. **(A)** Crypt histopathology (H&E and PAS staining), **(B)** colon crypt depth (μm), and **(C)** number of PAS-positive cells per mm^2^ colonic mucosa; **(D)** Histology score. C and T refer to control- and HS pigs, respectively. The numbers 1, 7, 14, 21 indicate dates of sample collection. *p < 0.05, **p < 0.01 compared with each group.

Morphological observation of colons revealed that the mucosal layer of the control group was intact, with no epithelial cells shed. In contrast, sustained HS caused epithelial cell sloughing, vasodilation, and mucosal hyperemia in the colonic intestinal epithelium from day 7 onwards (Figure 3A). The crypt depth for the HS pigs became shallower and by day 14, it was significantly different from that of the controls (*P* < 0.05) (Figure 3B). In the HS pigs, the number of goblet cells in the mucosal tissue area was markedly lower than in control pigs; the difference between groups by day 7 was significant (*P* < 0.05) (Figure 3C). The number of immune cells between the epithelial cells was also greater in the HS than the control pigs. The histology score was significantly increased (*P* < 0.01) on day 7, 14, and 21 in heat stressed pigs compared with each control group, and reached the maximum on day 7(Figure 3D).

### Effects of Heat Stress on Microbial Diversity in Pigs

**Figure 4:**
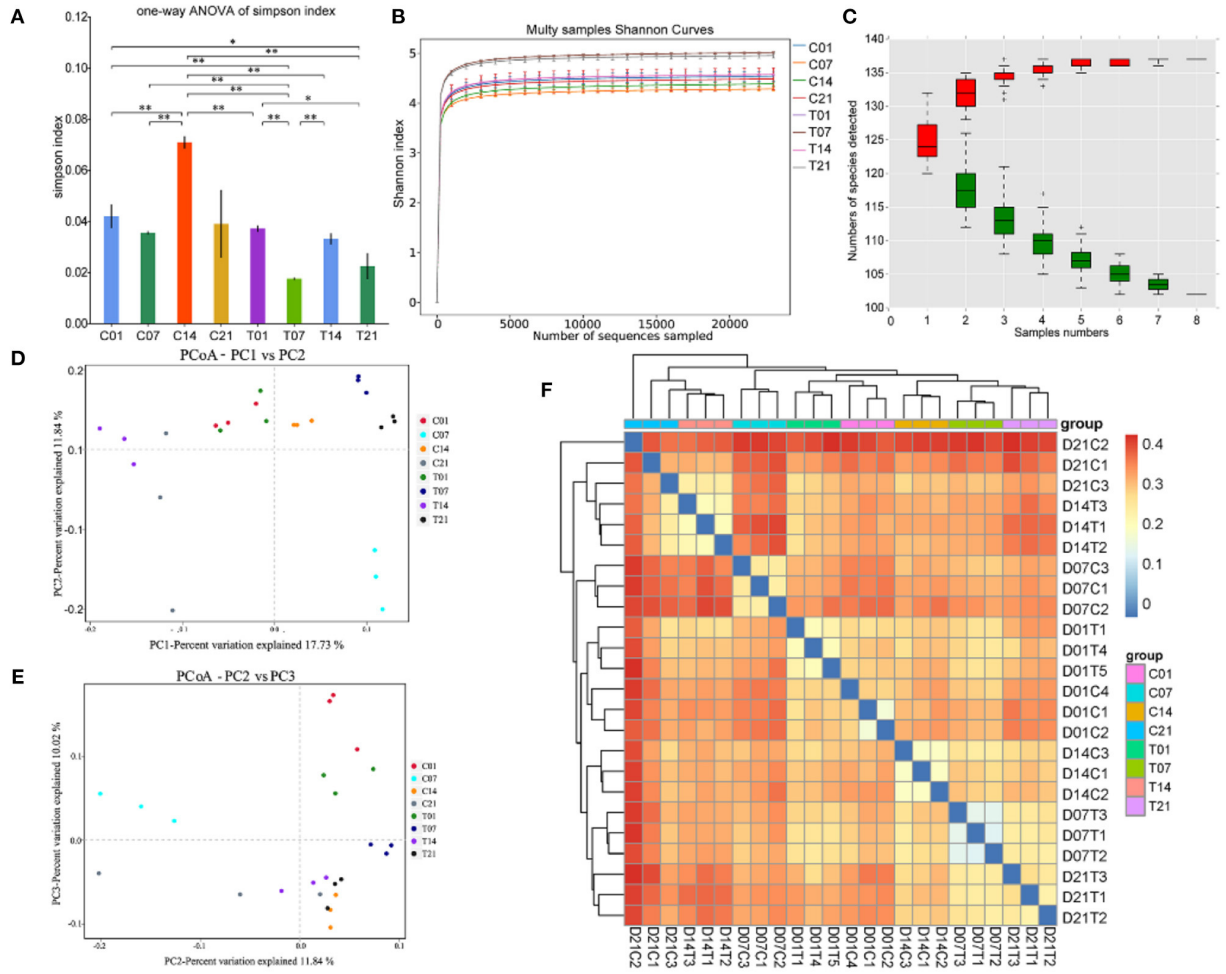
Effects of heat stress on pig colonic microbiome. **(A)** Simpson Index plot; **(B)** Multy Shannon curves; **(C)** Species accumulation curves; **(D,E)** Principle component analysis (PCoA); **(F)** Heatmap of sample distance. C and T refer to control- and HS pigs, respectively; 1, 7, 14, and 21 refer to sampling days of pig feces. ^*^P < 0.05, ^**^P < 0.01 compared with each group.

The Illumina HiSeq and paired-end methods were used to sequence the 16S and 18S rRNA constructs. The Simpson index values were lower in the HS group than that for the control group (Figure 4A). The Shannon curves plot disclosed that for each sample the curve was flat. Thus, there was sufficient sequencing data and the number of OTU species did not increase with sequencing quantity (Figure 4B). Each sample had numerous OTUs and was rich in species (Figure 4C). The PCoA indicated that the samples in the control group were highly similar and equidistant, whereas those of the HS group had relatively greater similarity (Figures 4D,E). The heatmap of sample distances shows clustering within groups. On day 14, the HS group was significantly different from the other groups. Compared with the control group, the change after day 1 of heat stress was not significant (Figure 4F).

### Effect of Heat Stress on Microbial Structure in Pigs

**Figure 5:**
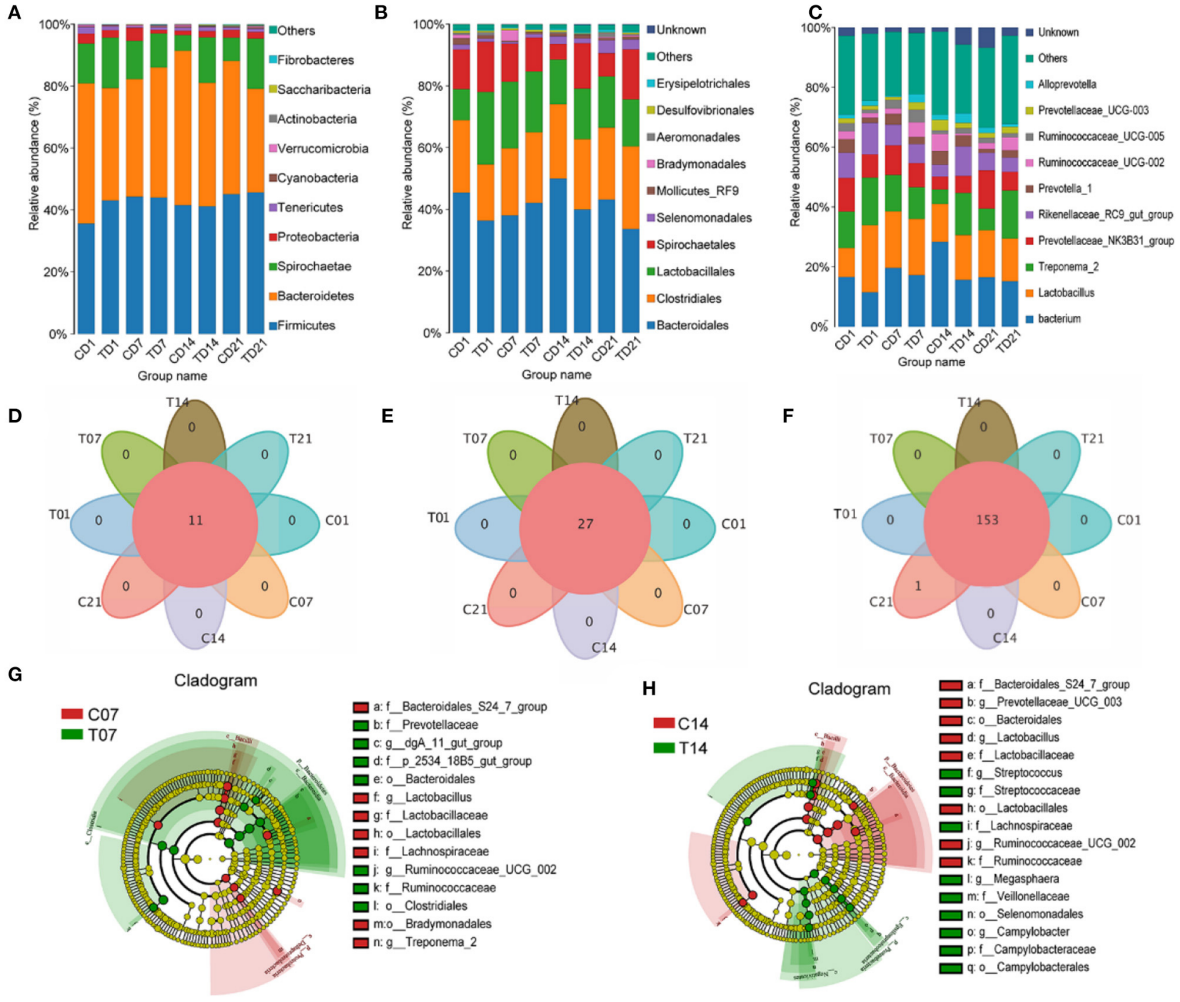
Effects of heat stress on pigs' colonic species composition. **(A)** Intestinal microbiota by phylum; **(B)** Intestinal microbiota abundance by order; **(C)** Intestinal microbiota abundance by genus; **(D)** Venn analysis by phylum; **(E)** Venn analysis by class; **(F)** Venn analysis by genus; **(G)** LEfSe analysis - day 7; **(H)** LEfSe (Line Discriminant Analysis Effect Size) analysis - day 14. C and T refer to control and HS pigs respectively; 1, 7, 14, and 21 refer to sampling days for pig feces and mouse transplantation of pig feces. Only the results with an LDA (Line Discriminant Analysis) significant threshold of > 4 are shown.

At the phylum level, the abundance of *Firmicutes* had increased on days 7 and 14 in the HS group, and the numbers of *Bacteroidetes* and *Spirochaetes* were lower on day 7, compared with those in the control group; on day 14, *Proteobacteria* were significantly more abundant in the HS group. *Cyanobacteria* and *Actinobacteria* had higher abundances on day 7 and day 14 in the HS group compared with numbers in the control group, while *Verrucomicrobia* and *Saccharibacteria* showed the opposite trends (Figure 5A). *Bacteroidales* and *Clostridiales* numbers were higher on days 7 and 21 in the HS group but lower on days 1 and 14 compared with those for each control group. *Lactobacillales* numbers were lower on days 7, 14, and 21 in the HS groups. *Spirochaetales* numbers were significantly lower on days 1, 7, and 21 in the HS pigs as compared to the control, but were higher on day 14. The abundance of *Selenomonadales* gradually increased in the HS groups and that of *Mollicutes RF9* greatly increased on days 1 and 14 in the HS groups compared with levels in each control group. *Campylobacterales* numbers were increased in all HS groups, especially on day 14 (Figure 5B).

At the genus level, within *Firmicutes*, it was mainly the numbers of *Lactobacilli* and *Ruminococcaceae* that changed. By day 14, the numbers of *Lactobacillus* and *Ruminococcaceae* had slightly declined. Significant changes in the numbers of *Spirochaetae* were reflected in *Treponema 2*. Among the *Bacteroidetes*, the numbers of *Alloprevotella, Rikenellaceae RC9 gut group*, and *Prevotellaceae* increased. Among the *Spirochaetes*, the numbers of Gram-negative *Treponema* significantly increased. The numbers in the genus *Bacteroides* diminished because of a decline in the number of *Prevotellaceae* (Figure 5C).

Venn analyses showed no differences at the phylum (Figure 5D) and order levels (Figure 5E) between groups, and at the genus level the difference from the control group was only significant on day 21 (Figure 5F). The LEfSe analysis showed that the relative differences in microbiome between the HS and control pigs on day 7 reflected increases in *Prevotellaceae, Clostridiales, Bacteroidales, Ruminococcaceae UCG 002*, and *dgA 11 gut group*, and on day 14 reflected increases in the number of opportunistic pathogens such as *Campylobacterales, Selenomonadales, Veillonellaceae, Lachnospiraceae*, and *Megasphaera* (Figures 5G,H).

### Correlation Analysis of Heat Stress on Microbiota Composition in Pigs

**Figure 6:**
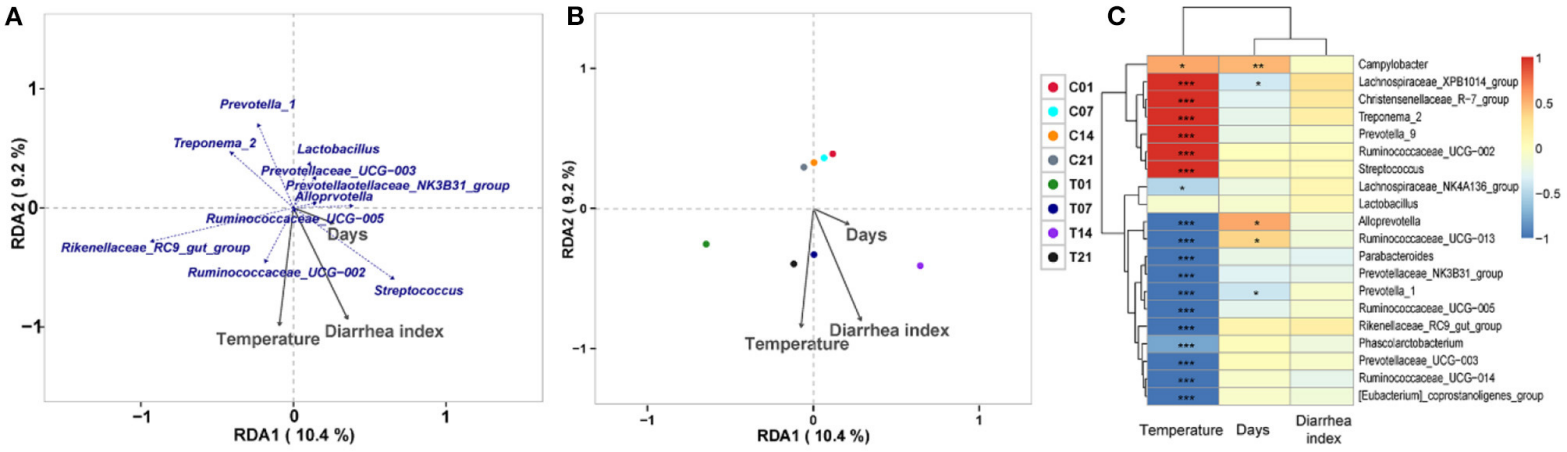
Correlation analysis of environmental factors on microbiota composition in pigs. **(A)** RDA (Redundancy analysis) / CCA (Canonical Correspondence analysis) analysis; **(B)** Distance-based redundancy analysis; **(C)** Relevance heatmap.

RDA/CCA analysis based on binary-Jaccard distances revealed that *Streptococcus, Prevotellaceae UCG*−*003*, and *Ruminococcaceae UCG*−*002* showed positive correlations with treatment, while *Prevotella 1, Prevotellaceae NK3B31 group, Alloprevotella, Ruminococcaceae UCG*−*005, Rikenellaceae RC9 gut group*, and *Treponema 2* showed opposing trends. The *Ruminococcaceae UCG*−*002, Streptococcus*, and *Prevotellaceae NK3B31 group* showed positive correlations with temperature, and *Prevotellaceae UCG*−*003, Lactobacillus, Prevotella 1*, and *Treponema 2* showed negative correlations with temperature. The *Ruminococcaceae UCG*−*002, Streptococcus*, and *Prevotellaceae NK3B31* group showed positive correlations with DI, while *Prevotellaceae UCG*−*003, Lactobacillus, Prevotella 1, Treponema 2, Rikenellaceae RC9 gut group*, and *Ruminococcaceae UCG*−*005* showed negative correlations (Figure 6A). Binary-Jaccard distance-based redundancy analysis (Figure 6B) and the Mantel Test (Table 2) showed that the HS treatment had a significant effect on microbiota formation (*P* < 0.05) and influenced the DI (*P* < 0.05). The relevance heatmap revealed that temperature had a significant effect on *Campylobacterales, Treponema2, Ruminococcaceae UCG-002, Alloprevotella*, and *Lactobacillales. Campylobacterales, Alloprevotella*, and *Prevotella 1* were significantly correlated with processing time (Figure 6C).

**Table 2:** Mantel test of environmental factors in pig microbiome.

**Environmental factors**	**Mantel test R statistic**	***P*-value**
Temperature	0.1290	0.021
Days	0.2630	0.003
Diarrhea index	0.4264	0.001

### Histopathology of Mouse Colonic Mucosa Following FMT

**Figure 7:**
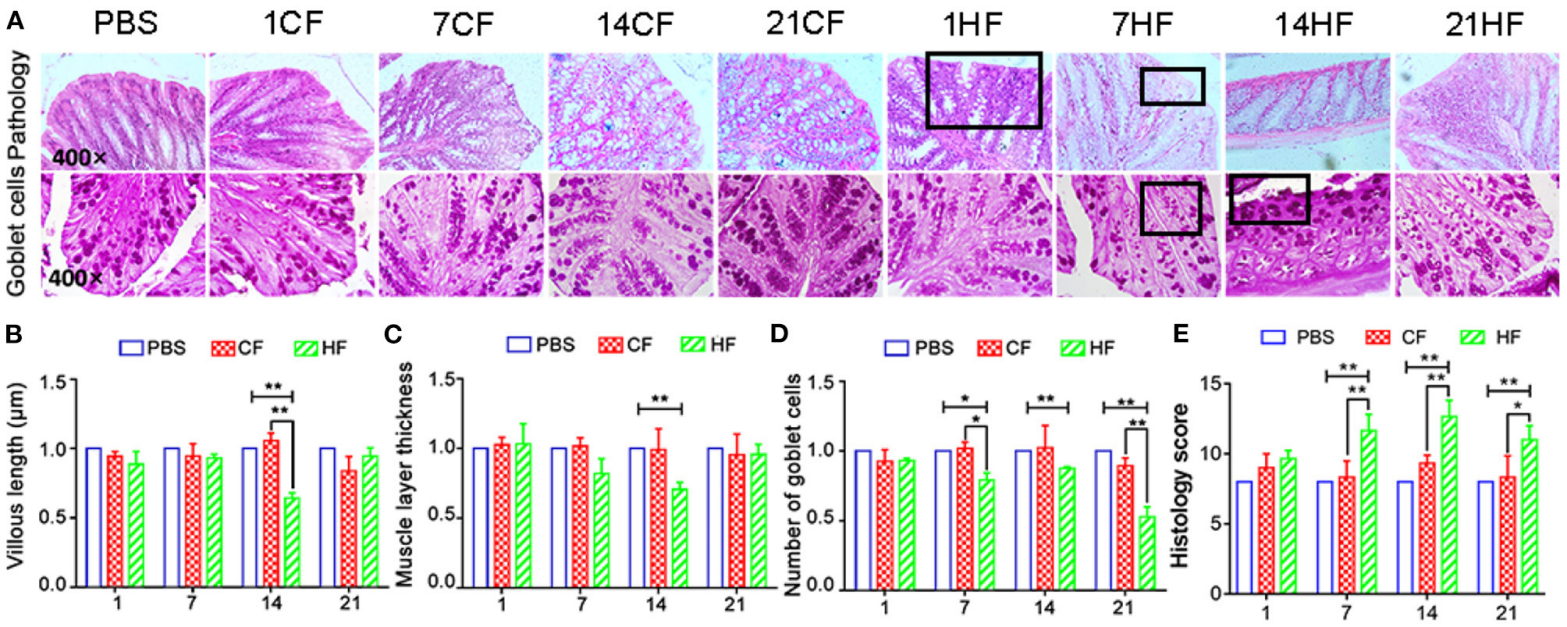
Histopathology of colonic mucosa of mice following pig fecal microbial transplantation. **(A)** H&E and PAS staining; **(B)** colonic mucosal height; **(C)** colon muscle layer thickness; **(D)** number of PAS-positive goblet cells per mm^2^ colonic mucosal; **(E)** Histology score of mice colon. CF & HF refer to mice administered with feces from control– and HS pigs respectively; 1, 7, 14, and 21 refer to sampling days of pig feces and mouse transplantation. ^*^P < 0.05, ^**^P < 0.01 compared with each group.

Following FMT, lymphocyte infiltration and tissue gaps were observed in the colonic mucosa of the HF1, HF7, and HF21 mice (Figure 7A). Goblet cell and mucosal epithelial shedding were comparatively lower in the HF14 group than in the controls (Figures 7A,D). Compared with the controls, the colonic mucosa was significantly shorter (*P* < 0.05) (Figure 7B), and the intestinal muscle layer was significantly thinner in the HF14 mice (*P* < 0.05) (Figure 7C). Comparing with PBS and each CF group, the histology score were increased significantly (*P* < 0.05) in colon of mice that fed the HS-pigs feces by day 7, 14, and 21 (Figure 7E).

### FMT Effects on Mouse Microbial Diversity

**Figure 8:**
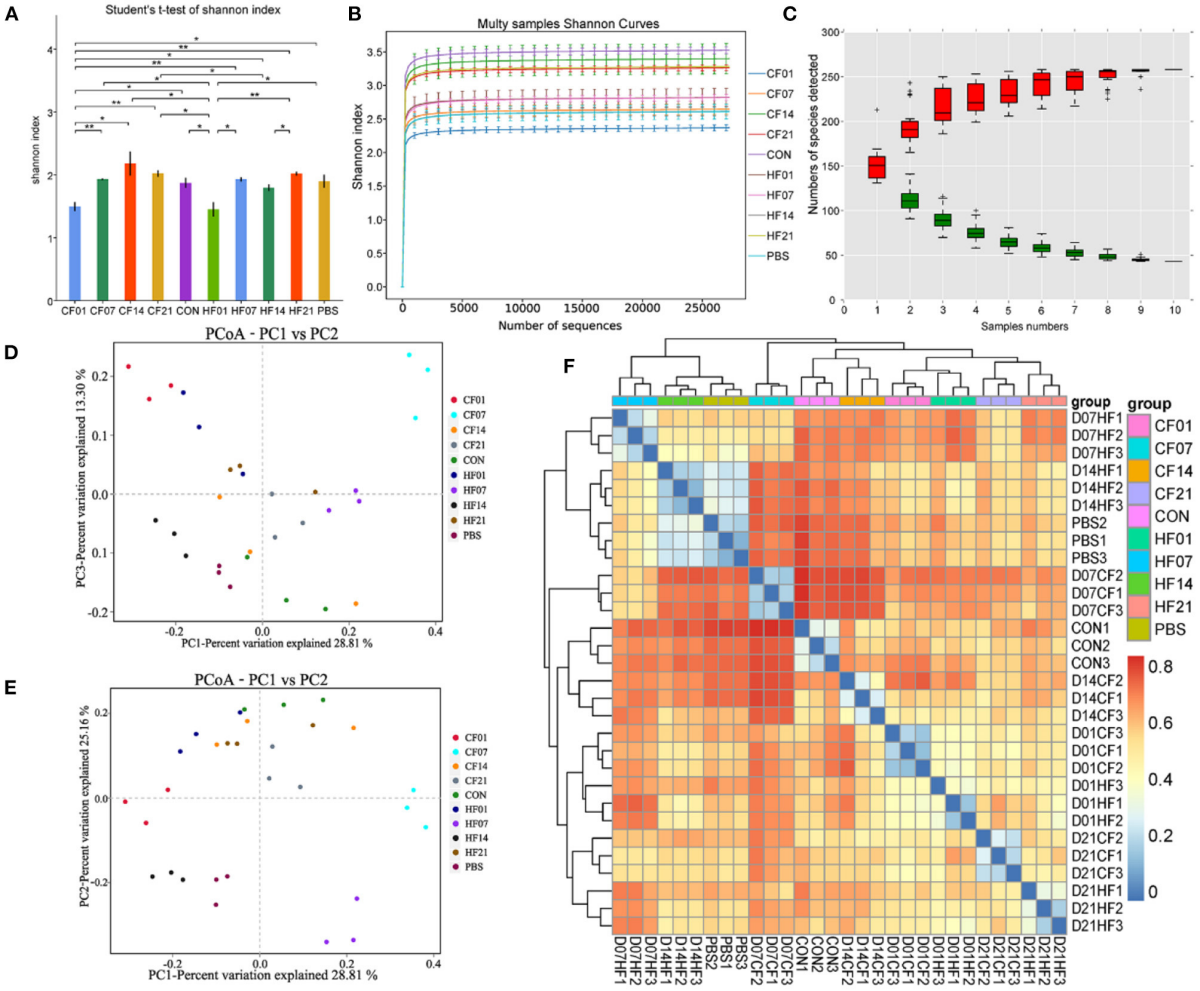
Effects of heat stress on mice colonic microbiome. **(A)** Shannon Index plot; **(B)** Multy Shannon curves; **(C)** Species accumulation curves; **(D,E)** PCoA; **(F)** Heatmap of samples distance. CF & HF refer to infusion with feces from control– and HS animals, respectively; 1, 7, 14, and 21 refer to sampling days of pig feces and mouse transplantation. CF & HF refer to mice administered with feces from control– and HS pigs respectively. ^*^P < 0.05, ^**^P < 0.01 compared with each group.

The Shannon curves (Figure 8A) analysis showed that alpha diversity was significantly (*P* < 0.1) changed after FMT. The Shannon curves plot disclosed that for each sample the curve was flat (Figure 8B). Thus, there was sufficient sequencing data and the number of OTU species did not increase with sequencing quantity (Figure 8C). Each sample had numerous OTUs and was rich in species. Most species were detected in the samples. The PCoA indicated that the samples in the control group were highly similar and equidistant, whereas those of the FMT (CF & HF) group had relatively greater similarity (Figures 8D,E). The heatmap of sample distances shows clustering within groups (Figure 8F).

### FMT Effects on Mouse Colonic Microbiome

**Figure 9:**
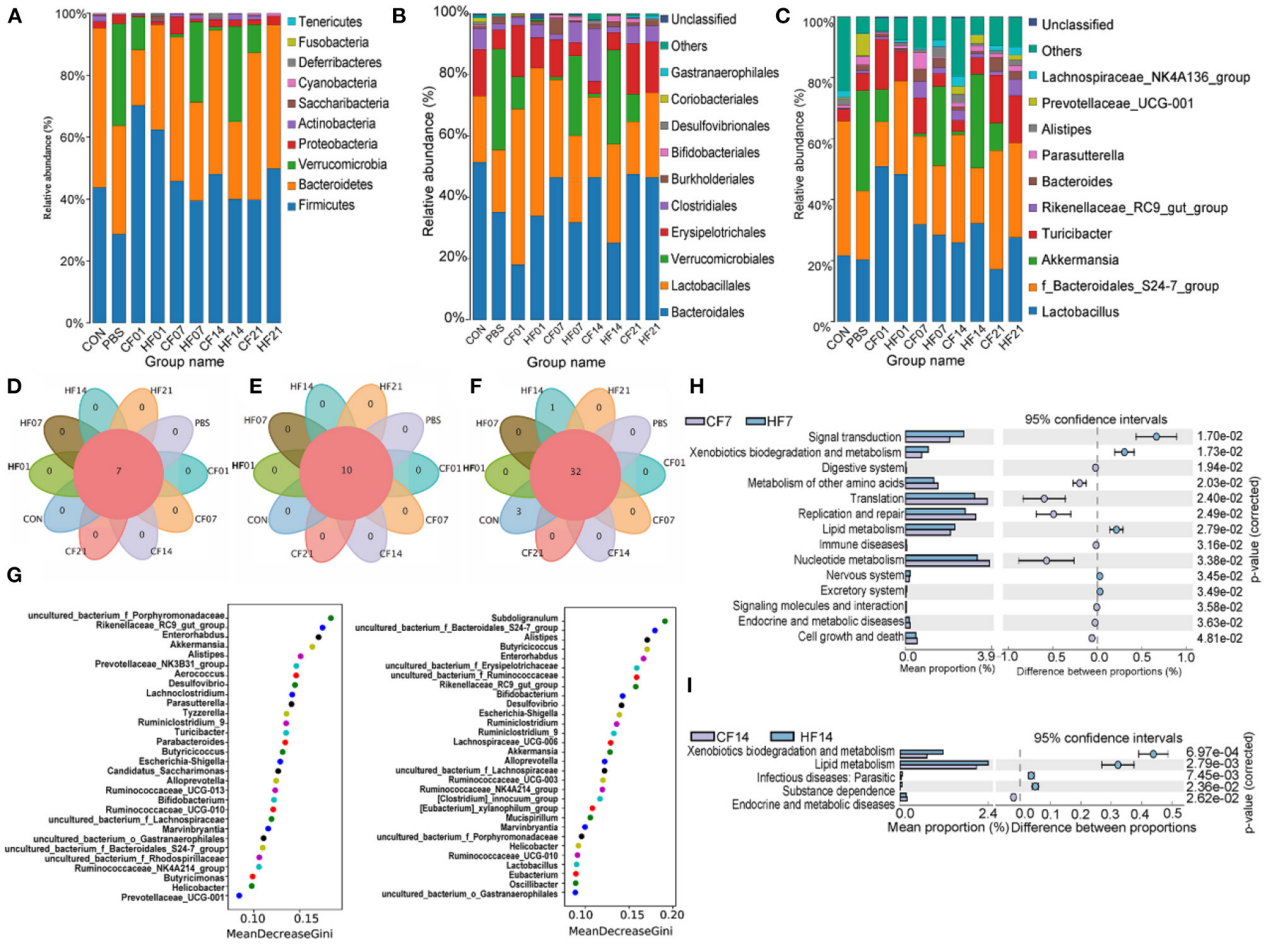
Effects of heat stress on mice colonic species composition. **(A)** Intestinal microbiota structure by phylum; **(B)** Intestinal microbiota structure by class; **(C)** Intestinal microbiota structure by genus; **(D)** Venn analysis by phylum; **(E)** Venn analysis by class; **(F)** Venn analysis by genus; **(G)** Random forest distribution analysis on day 7 and day 14; **(H)** KEGG functional difference prediction (CF7 vs. HF7); **(I)** KEGG functional difference prediction (CF14 vs. H1F4). CF & HF refer to infusion with feces from control– and HS animals, respectively; 1, 7, 14, and 21 refer to sampling days of pig feces and mouse transplantation.

At the phylum level, compared with the PBS group, the abundance of *Firmicutes* was higher in all FMT (CF & HF) groups, except for on day 21 in the CF group. *Bacteroidetes* was more abundant on days 7 and 14 in the CF group, comparing with each HF group. *Verrucomicrobia* numbers were lower in all FMT (CF & HF) groups than in the PBS group. *Proteobacteria* abundance was lower in all FMT (CF & HF) groups, except for on day 7 in the CF group. Compared with numbers for each CF group, *Firmicutes* numbers were lower on days 1, 7, and 14 in HF groups, and significantly (78.44%) higher on day 21. *Bacteroidetes* and *Proteobacteria* numbers were higher on day 1, but lower on days 7 and 14. The results for *Verrucomicrobia* were in contrast with those for *Bacteroidetes (*Figure 9A).

At the order level, compared with numbers for the PBS group, *Bacteroidales* abundance was higher on days 7, 14, and 21 in the CF groups, but lower on days 1, 7, and 14 in the HF groups, and significantly on day 1 in the CF group. *Lactobacillales* abundance was higher in all FMT (CF & HF) groups than in the PBS group, except for in the CF group on day 21. *Verrucomicrobiales* abundance was lower in all FMT (CF & HF) groups and *Erysipelotrichales* was higher on days 1, 7, and 21 in the CF group and on days 1 and 21 in the HF groups. *Clostridiales* abundance was significantly (15.2%) higher on day 7 in the CF groups compared with levels in the PBS group. Compared with numbers in the CF group, the abundance of *Bacteroidales* was lower on days 7 and 14 in the HF groups, while the abundance of *Verrucomicrobiales*, in contrast, was significantly higher (25.03% on days 7, 29.64% on days 14). *Lactobacillales*, as well as *Erysipelotrichales*, were more abundant on days 1 and 7 in the HF groups and less abundant on days 14 and 21, while the results for *Clostridiales* showed the opposite trend (Figure 9B).

At the genus level, compared with the numbers for the PBS group, *Lactobacillus* was more abundant in all FMT (CF & HF) groups, except for in the CF group on day 21. Uncultured bacterium of the *Bacteroidales S24-7 group* were more abundant in the CF group on days 7, 14, and 21 and less abundant on day 14 in the HF group. *Akkermansia* numbers were lower in all FMT (CF & HF) groups and *Turicibacter* was more abundant in the CF groups, except for on day 14, and was less abundant in the HF groups on days 7 and 14. *Rikenellaceae RC9 gut group* abundance was higher on days 7, 14, and 21 in the CF and HF groups. Compared with levels in the HF groups, *Lactobacillus* and *Rikenellaceae RC9 gut group* were less abundant on days 1 and 7 in the HF group and more abundant on days 14 and 21. *Uncultured bacterium f_Bacteroidales_S24-7 group* showed contrasting results. The abundance of *Akkermansia* was significantly (25.03%, 29.64%) higher on days 7 and 14 in the HF groups than in the PBS group, and significantly lower (−10.59%, −9.03%) on days 1 and 21. *Turicibacter* abundance was higher on day 14 in all HF groups (Figure 9C). Venn analyses showed that there were no differences between the phylum (Figure 9D), order (Figure 9E), and genus levels (Figure 9F) between samples. Random forest analysis of microbiota on day 7 showed the top five genera were uncultured bacterium *f Porphyromonadaceae, Rikenellaceae RC9 gut group, Enterorhabdus, Akkermansia*, and *Alistipes*, and on day 14 were *Subdoligranulum, uncultured bacterium f Bacteroidales S24-7 group, Alistipes, Butyricicoccus*, and *Enterorhabdus* (Figure 9G). The KEGG plot revealed relative differences in lipid metabolism and xenobiotic biodegradation and increases in signal transduction for HF7 and HF14 (Figures 9H,I).

### Correlation Analysis of FMT on Microbiota Composition in Mice

**Figure 10:**
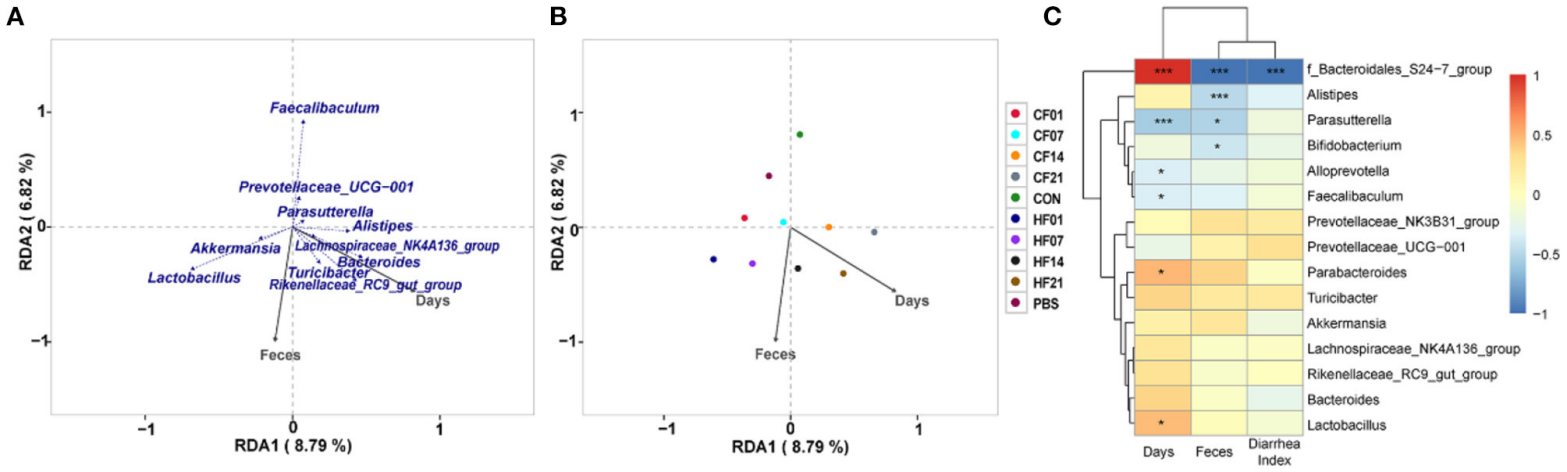
Correlation analysis of environmental factors on microbial species composition in mice. **(A)** RDA (Redundancy analysis) / CCA (Canonical Correspondence analysis) analysis; **(B)** Distance-based redundancy analysis; **(C)** Relevance heatmap. ^*^P < 0.05, ^***^P < 0.001 compared with each group.

RDA/CCA analysis based on binary-Jaccard distances revealed that *Lactobacillus, Akkermansia, Turicibacter, Rikenellaceae RC9 gut group*, and *Bacteroides* showed positive correlations with the date FMT, and the results for *Parasutterella, Faecalibaculum*, and *Prevotellaceae UCG*−*001* showed the opposite relationships. *Lactobacillus, Akkermansia, Faecalibaculum*, and *Prevotellaceae UCG*−*001* showed positive correlations with date of FMT, and *Turicibacter, Rikenellaceae RC9 gut group*, and *Bacteroides* showed negative correlations (Figure 10A). Binary-Jaccard distance-based redundancy analysis (Figure 10B) and Mantel Test (Table 3) results showed that the FMT treatment significantly affected microbiota formation (P < 0.05). The results of the relevance heatmap showed that *uncultured bacterium f Bacteroidales S24-7 group, Alistipes, Parasutterella*, and *Bifidobacterium* were significantly affected by FMT (Figure 10C).

**Table 3:** Mantel test of environmental factors in mice microbiome.

**Environmental factors**	**Mantel test R statistic**	***P*-value**
Graft	0.2552	0.005
Date of graft	0.0772	0.087

## Discussion

Stress refers to the sum of non-specific responses produced by the body's non-specific, abnormal stress factor stimulation inside and outside, an integral part of the body's regulation, feedback, adaptation and protection mechanisms (52). In the pathogenesis of inflammation in the colon, both genetic predisposition and environmental factors are important in promoting inappropriate host immune responses to the intestinal microbiome. The role of stress-induced gut mucosal pathophysiology has not been fully elucidated. Under HS, the serum COR content and HSP70 expression of tissues increases. Gabler et al. found that HS significantly reduced pig performance and intestinal integrity and increased blood endotoxin concentrations. Our results showed that the anal temperature, diarrhea, and the expression of heat shock proteins (HSP27 and HSP70) were significantly increased in pigs under heat stress. Increase in the cortisol and endotoxin concentrations of pigs' peripheral blood indicate that heat stress can affect intestinal inflammation and damage function in pigs. We also found a decrease in stress response such as recovery of expression of HSPs, intestinal concentrations of LPS and COR at 21 days, which may be the adaptive resistance of heat-stressed pigs to hyperthermic environments (53), the pigs' metabolism is altered to reduce heat production and alleviate the stress response under.

In pigs, HS diverts blood to the periphery to maximize radiant heat dissipation. This response causes vasoconstriction in the gastrointestinal tract and hypoxia in the intestinal epithelium because of the reduced blood supply, resulting in a reduction in nutrient flow and compromised intestinal integrity and function (54). Pearea et al. found that exposure to 37°C for 4 h damaged the intestinal mucosa of growing pigs. After 6 h of heat stress (37°C), large numbers of intestinal epithelial cells were sloughed, and severe submucosal congestion and edema was observed. The HS pigs in the present study showed shorter intestinal mucosal, and shallower crypts at days 7 and 14 of HS groups, comparing with the control groups. Other stressors, such as psychosocial events in humans cold stress, and restraint stress in mice induced strong inflammatory responses in the gut. Therefore, HS-induced vasoconstriction and hypoxia in the gastrointestinal tract do not fully explain IBD in pigs, and another mechanism may be involved in this pathogenesis. Le Sciellour et al. (55) found that acute and chronic heat-stressed pigs exhibited significant changes in the gut microbiome in relation to body temperature, which is consistent with our study. Xiong et al. (56) found alterations in intestinal microbiota coincide with impaired intestinal morphology and dysfunctional intestinal inflammations in growing-finishing pigs under constant chronic HS, which indicates that gut microbes contribute to the development of IBD, and the results are similar to our findings.

The epithelial mucosa comprises the first-line protective barrier can effectively prevent the invasion of opportunistic pathogens (57, 58). Diarrhea increases at day 6 with a maximum at day 14, when the numbers of opportunistic bacterial pathogens such as *Campylobacterales, Veillonellaceae*, and *Megasphaera* increased in the intestinal microbiome. Interestingly, we found that the concentration of *Clostridium sensu stricto-1* was lower after HS. Oxidative stress is associated with host heat or cold stress and may generate reactive oxygen species (ROS). The bacteria residing on the colonic mucosa have a relatively greater oxygen tolerance, which may favor the proliferation of aerotolerant phyla, such as *Actinobacteria* and *Proteobacteria*, in the gut. In contrast, gut microbiota can directly or indirectly contribute to ROS production via the mucosal cells (59). *Helicobacter pylori* generates ROS and also triggers neutrophils to produce them, (60). *Helicobacter pylori* also enhances nitric and nitrous oxide production by activating macrophages (61). Correlation analysis showed that microbial composition changes were more significant as the heat stress treatment continued.

Pseudo germ-free animals are often used to study the effect of FMT on the host (62, 63) It is reported that presence of gut microbiota predisposes mice to IBD relative to germ-free animals (14). And DSS-Induced Ulcerative Colitis in Pseudo Germ-Free mice model can more help study the inter-reactive mechanism of intestinal microbiota and host. (64) FMT alters bacterial composition and establishes trans-kingdom equilibrium between gut fungi, viruses, and bacteria, to promote the recovery of microbial homeostasis. FMT is not a one size fits all therapy and further studies are required to identify intestinal microbial components that have specific effects in patients with different gut-related diseases (65). The intestinal microbial composition changed in the HF mice following FMT administration of pig feces.

Therefore, an inflammatory response had developed in the intestines of the FMT-treated HF mice. The changes in LEfSE analysis and the KEGG plot revealed relative differences in lipid metabolism and xenobiotic biodegradation and increases in signal transduction on days 7 and 14, showing that the potential contact with intestinal microbial composition changed after transplanting heat-stressed pigs' feces. Breitruck et al. reported that the structure of intestinal mucosa is damaged in mice with IBD. Diao et al. reported that gut microbiota affected certain pig traits and transferred their phenotypes to mice receiving pig feces via a different mechanism. The gut microbiota also influenced epithelial cell morphology and renewal rates, intestinal nutrient digestion and absorption, and the gut barrier (66, 67). Pseudo-sterile mouse models are often used to study the interaction between the intestinal flora and the host. Using broad-spectrum antibiotics was a common method to cause a bacterial imbalance, reduce the abundance and diversity of intestinal microorganisms to establish the pseudo-sterile model (68, 69). Interestingly, in our study the construction of a pseudo-sterile mouse model resulted in an increase in *Akkermansia* in the colon but after FMT, the abundance of *Akkermansia* was lower, indicating a negative impact of FMT in mice. The numbers of opportunistic bacterial pathogens such as *Turicibacter* were higher on day 14 in the HF groups, indicating potentially harmful effects of FMT with high HS intensity in mice. Correlation analyses showed that microbial composition changes were associated with FMT from pigs and had a negative impact on the HF group on day 7, such as fewer goblet cells, shorter colonic mucosal, and reduction of *Lactobacillus*. Although the molecular mechanisms require further investigation, this study has provided new insights into the dysbacteriosis of porcine colon microbiota caused by heat stress to promote the development of IBD.

## Conclusion

The present study showed that HS-induced intestinal dysbiosis disrupted gut microbiome composition and increased the numbers of opportunistic, pathogenic Gramnegative bacteria. This process compromised the colonic inflammation and damage, reproduced in mice following FMT. This study enhances our understanding of stressinduced inflammation in the colon and the diarrhea increase in mammals subjected to prolonged HS.

## Data Availability Statement

The original contributions presented in the study are publicly available. This data can be found here: https://www.ncbi.nlm.nih.gov/sra/PRJNA729581 and https://www.ncbi.nlm.nih.gov/sra/PRJNA729473.

## Ethics Statement

The experimental protocols involving the management and care of pigs and mice were approved by the Animal Care and Use Committee of Guangdong Ocean University, Zhanjiang, China (Permit No. 206-1108).

## Author Contributions

XJ conceived the project and designed the experiments. CH, YY, DG, TY, JL, YP, and LW conducted the experimental work and analyzed the data. XJ and RG interpreted the results. CH and YP prepared the figures and wrote the manuscript. XJ, RG, HY, and JC edited the manuscript. XL, XM, and ZY participated in the enrichment analysis and manuscript writing and revision. All authors read and approved the manuscript content.

## Funding

This study was supported by the National Natural Science Foundation of China [grant numbers 31472243, 31902314]; Natural Science Foundation of Guangdong Province, China [grant number: 2019A1515011142]; the Project of Enhancing School with Innovation of Guangdong Ocean University [grant number: GDOU230419057]; the Basic Research Project of Shenzhen Science and Technology Innovation Commission (JCYJ20190813142005766).

## Conflict of Interest

The authors declare that the research was conducted in the absence of any commercial or financial relationships that could be construed as a potential conflict of interest.

## Publisher's Note

All claims expressed in this article are solely those of the authors and do not necessarily represent those of their affiliated organizations, or those of the publisher, the editors and the reviewers. Any product that may be evaluated in this article, or claim that may be made by its manufacturer, is not guaranteed or endorsed by the publisher.

## Abbreviations

BW: body weight
CCA: Canonical Correspondence analysis
COR: Cortisol
FMT: Fecal microbial transplantation
HSP27: Heat shock protein 27
HSP70: Heat shock protein 70
HS: Heat Stress
IBD: Inflammatory bowel disease
IL: Interleukin
KEGG: Kyoto Encyclopedia of Genes and Genomes
LEfSe: line discriminant analysis effect size
LPS: Lipopolysaccharide
NF-κB: Nuclear factor κ appa B
OUT: Operational taxonomic unit
PBS: Phosphate buffer saline
RDA: Redundancy analysis
SPF: Specific Pathogen Free.
