# Supplementary material for: Heat Stress-Induced Dysbiosis of Porcine Colon Microbiota Plays a Role in Intestinal Damage: A Fecal Microbiota Profile
Source: Front Vet Sci. 2022 Mar 1;9:686902. doi: 10.3389/fvets.2022.686902 (PMC8921775; doi:10.3389/fvets.2022.686902)
Supplement: Supplementary file 1 [file Data_Sheet_1.DOCX]

**Supplementary material**


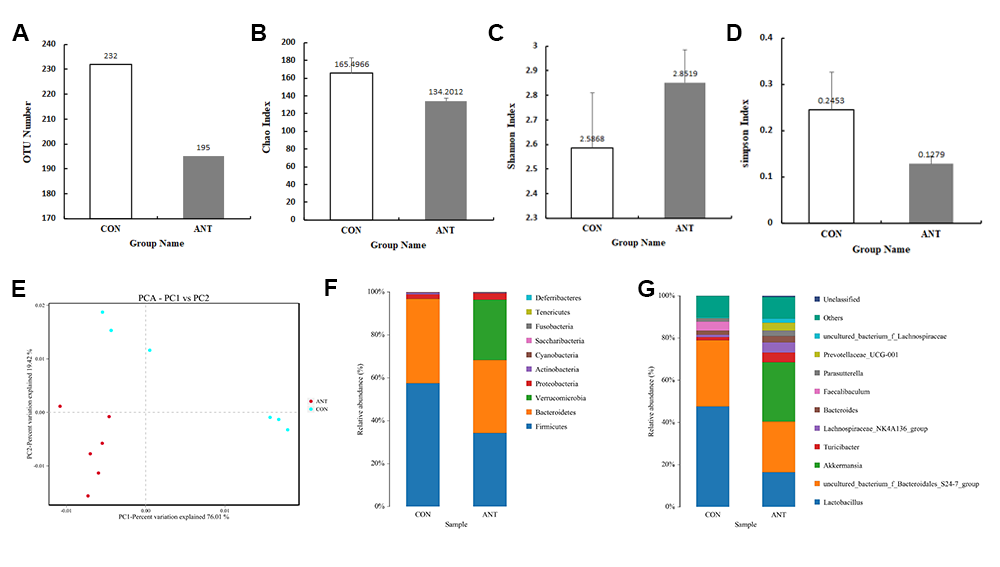


Figure S1. Effect of mixed antibiotics feeding on colonic microbiome of mice

CON: without treatment; ANT: mixed antibiotics feeding.

1. OUT number of samples; (B) Chao Index plot; (C) Shannon Index plot; (D) Simpson Index plot; (E) PCoA analysis; (F) Intestinal flora structure by phylum; (G) Intestinal flora structure by genus, * indicates significance at p < 0.05 compared with the control group, ** indicates significance at p < 0.01 compared with the control group.

Following mixed antibiotics feeding:

1. Compared with control group, OTU number of ANT decreased significantly (Fig. S1A).
2. Compared with the control group, alpha diversity analysis of Chao and Simpson indexes decreased significantly while Shannon Index increased (Fig. S1B, C & D), Thus the species richness and diversity of colonic microbiome decreased.
3. The principal component beta diversity analysis showed that compared with the control group, the distance between the samples after antibiotic treatment was closer (Fig. S1E).
4. When the intestinal flora structure by phylum was compared with the control group, antibiotic feeding increased the abundance of *Verrucomicrobia* but reduced the abundance of *Firmicutes* and *Bacteroides* which accounted for 96.78% in the control group. *Firmicutes*, *Bacteroides* and *Verrucomicrobia* accounted for 96.40% in the ANT group. The abundance of *Proteobacteri*a increased and the abundance of *Actinobacteria* decreased. (Fig. S1F).
5. At the genus level, compared with the control group, the antibiotics significantly reduced the abundance of *Lactobacillus*, *Bacteroidales S24-7 group*, and *Faecalibaculum*, and increased the *Bacteroides*, *Turicibacter*, *Akkermansia*, and *Lachnospiraceae NK4A136 group.* (Fig. G).
